# Supplementary figures and images for: Induced pluripotent stem cell-derived human macrophages as an infection model for Trypanosoma cruzi
Source: PLoS Negl Trop Dis. 2025 Oct 24;19(10):e0012987. doi: 10.1371/journal.pntd.0012987 (PMC12622848; doi:10.1371/journal.pntd.0012987)

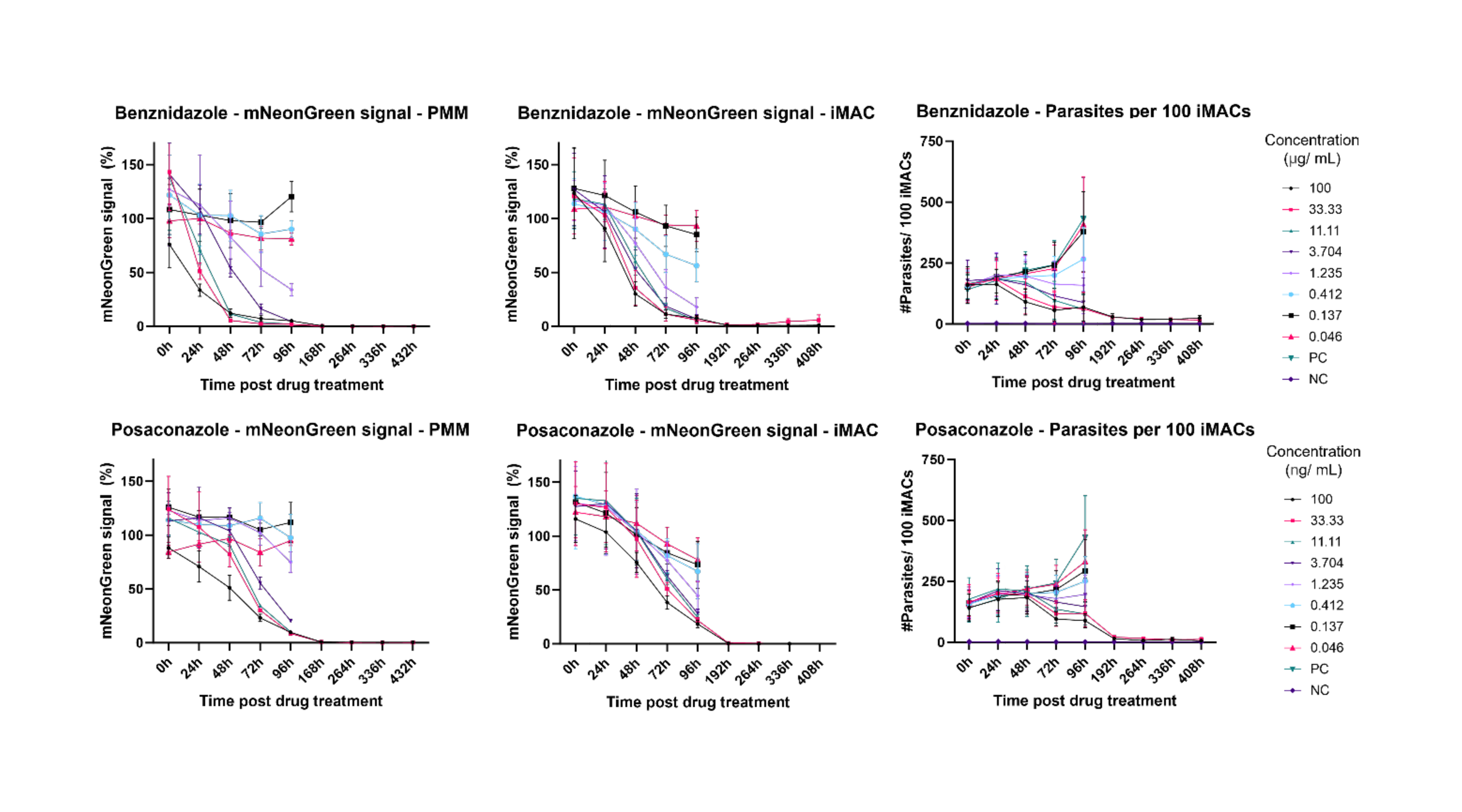

Supplement: S1 Fig — The response was measured as the total mNeonGreen signal and the number of parasites per 100 cells. A negative, uninfected control and a positive, infected but untreated control were included. The mNeonGreen signal is presented as percentage, corrected by subtracting the negative control and normalised by a positive control. Starting at 192 h, only the highest drug concentrations are shown as massive cell death due to parasite infection occurred at lower concentrations and in the positive, untreated control. (TIF) [file pntd.0012987.s001.tif]
